# Hepatic Alveolar Echinococcosis: Predictive Biological Activity Based on Radiomics of MRI

**DOI:** 10.1155/2021/6681092

**Published:** 2021-04-09

**Authors:** Bo Ren, Jian Wang, Zhoulin Miao, Yuwei Xia, Wenya Liu, Tieliang Zhang, Aierken Aikebaier

**Affiliations:** ^1^Department of Imaging Center, The First Affiliated Hospital of Xinjiang Medical University, Li Yu Shan Road, No. 137 Urumqi City 830054, China; ^2^Huiying Medical Technology Co., Ltd., Room A206, B2, Dongsheng Science and Technology Park, HaiDian District, Beijing City 100192, China

## Abstract

**Background:**

To evaluate the role of radiomics based on magnetic resonance imaging (MRI) in the biological activity of hepatic alveolar echinococcosis (HAE).

**Methods:**

In this study, 90 active and 46 inactive cases of HAE patients were analyzed retrospectively. All the subjects underwent MRI and positron emission tomography computed tomography (PET-CT) before surgery. A total of 1409 three-dimensional radiomics features were extracted from the T2-weighted MR images (T2WI). The inactive group in the training cohort was balanced via the synthetic minority oversampling technique (SMOTE) method. The least absolute shrinkage and selection operator (LASSO) regression method was used for feature selection. The machine learning (ML) classifiers were logistic regression (LR), multilayer perceptron (MLP), and support vector machine (SVM). We used a fivefold cross-validation strategy in the training cohorts. The classification performance of the radiomics signature was evaluated using receiver operating characteristic curve (ROC) analysis in the training and test cohorts.

**Results:**

The radiomics features were significantly associated with the biological activity, and 10 features were selected to construct the radiomics model. The best performance of the radiomics model for the biological activity prediction was obtained by MLP (AUC = 0.830 ± 0.053; accuracy = 0.817; sensitivity = 0.822; specificity = 0.811).

**Conclusions:**

We developed and validated a radiomics model as an adjunct tool to predict the HAE biological activity by combining T2WI images, which achieved results nearly equal to the PET-CT findings.

## 1. Introduction

Hepatic alveolar echinococcosis (HAE) is a parasitic disease caused by the larvae of *Echinococcus multilocularis* that parasitize the liver [1]. HAE causes lesions that are infiltrative and may spread to distant regions of the body, impairing health and may cause death. In its initial stages, HAE is idiopathic; thus, most patients are diagnosed late, rendering them unsuitable for radical resection surgery as they already have large hepatic lesions with vascular or biliary structure involvement [2]. The only choice of treatment for patients who are not candidates for radical resection surgery and those undergoing palliative resection involves antihydatid therapy with drugs such as albendazole [3]. However, there is no well-defined treatment period for HAE, which leads to long-term medication-associated complications in patients [4, 5]. Theoretically, the critical indicator for medication termination involves the absence of biological activity of HAE lesions; however, this criterion is not clinically feasible. As a result, assessing the biological activity of the HAE lesions is vital for the selection and design of treatment methods, including antiechinococcal chemotherapy for patients before and after surgery. It is against this background that determining the state of HAE lesions, whether active or inactive, is the primary goal of imaging procedures in clinical practice.

Although the proliferation and biological activity of HAE lesions can be evaluated by CT perfusion, energy CT imaging, and diffusion-weighted MRI [6–9], PET-CT is the most prioritized method globally for assessing HAE lesions [10, 11]. However, compared with CT and PET-CT, MRI has the advantages of having no radiation and being noninvasive. Moreover, MRI shows better tissue contrast and shows the small vesicle structure of the lesions [6, 7]. Furthermore, MRI can detect small lesions in the early stage noninvasively and does not use radiation; therefore, MRI is used as the preferred imaging examination for HAE lesions in patients.

Research has shown that radiomics data analysis can provide vital quantitative imaging information to quantitatively and objectively analyze tumors and other lesions [12]. Accordingly, radiomics has been successfully applied in the diagnosis, treatment, and evaluation of multiple tumor types in the medical field [13–18]. Currently, radiomics research on HAE is in its early stages. This study is aimed at extracting high-throughput features through HAE lesion segmentation, dimensional reduction analysis, and training machine learning to establish prediction models for prognosis, diagnosis, and monitoring of HAE lesions in patients.

## 2. Materials and Methods

### 2.1. Data Collection

This was a retrospective study in a single institution, approved by the Medical Ethics Review Committee of the First Affiliated Hospital of Xinjiang Medical University, and exempted from informed consent. From January 2012 to June 2020, 156 patients with HAE were admitted and diagnosed at the First Affiliated Hospital of Xinjiang Medical University. In this study, the PET-CT findings were considered as the “gold standard” to assess whether the lesions have biological activity or not. After that, a predictive model based on MRI was constructed to predict the biological activity of HAE lesions as a basis for prognosis, diagnosis, and monitoring of HAE lesions in patients. HAE patients (confirmed by imaging and postoperative pathology) who underwent abdominal MRI scan and PET-CT examination (images were transferred to PACS), with no history of chronic liver disease, with no previous history of liver surgery, and with no primary solitary space-occupying lesion were included. Conversely, HAE patients whose MRI and PET-CT image quality were poor (*n* = 2); whose PET imaging results were lacking (*n* = 15); who had extensive fibrosis, nodules, or old lesions in the liver (*n* = 1); and who previously confirmed and were already treated by surgical intervention (*n* = 2) were excluded. As a result, 136 patients were enrolled in this study. According to the results of PET-CT, the patients were divided into the active group (90 cases) and the inactive group (46 cases).

### 2.2. Image Data Acquisition

In this study, MRI was performed using the Siemens 3.0 T (Skyra) or 1.5 T (Avanto) MR scanner with an 18/8-channel phased array body coil. All patients were asked to fast for about six hours before scanning, after which they underwent upper abdomen MRI examination in the supine position. All patients underwent MR imaging with T1-weighted, T2-weighted, and fat-suppressed T2-weighted image delineation. The MR imaging protocol was slightly adjusted due to different devices causing minor adjustments to the parameters. The MR scan sequences were as follows:

(1) 3.0 T
  - T1WI: TR/TE = 400/8.0 ms; FOV = 320 mm × 320 mm; matrix = 320 × 192; NEX = 2.0; ST = 3.0 mm
  - T2WI: TR/TE = 4000/125 ms; NEX = 4.0; slice thickness = 3.0 mm
(2) 1.5 T
  - T1WI: TR/TE = 200/4.5 ms; matrix = 204 × 256
  - T2WI: TR/TE = 3500/100 ms

After the acquisition of 3D data, the attenuation correction of the PET image was performed based on the CT image, and the corrected PET image was automatically fused with the CT image to obtain axial, coronal, sagittal, and PET-CT fusion images. Two nuclear medicine doctors with more than ten years of experience in the diagnosis of PET-CT examined the images. In case of disagreement, the negotiated results were considered. According to the information provided by the MRI images, the SUV value was measured at the corresponding position. For each patient, two independent examinations were performed and completed within a week.

### 2.3. Radiomics Workflow

**Figure 1:**
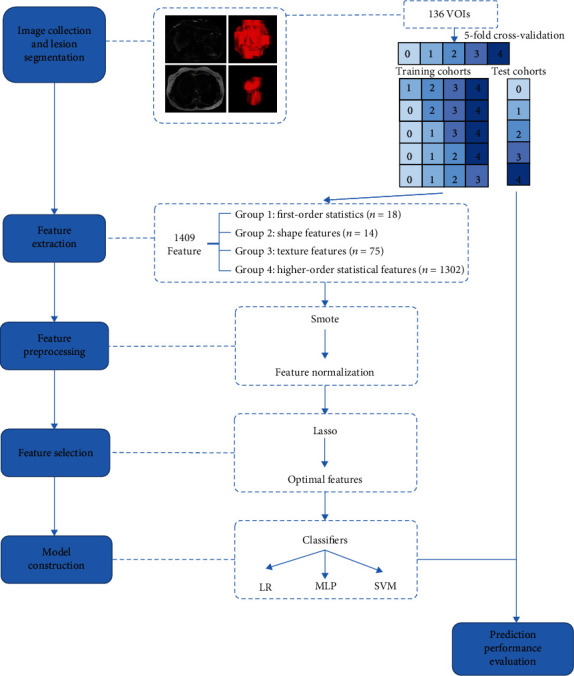
Illustration of the radiomics workflow adopted in this study. Note: SMOTE (synthetic minority oversampling technique); LASSO (least absolute shrinkage and selection operator); LR (logistic regression); MLP (multilayer perceptron); SVM (support vector machine).

Figure 1 illustrates the radiomics workflow adopted in this study. It included image collection; lesion segmentation; and radiomic feature extraction, selection of features, construction of models in the training cohorts, and evaluation of the performance of prediction models in the test cohorts.

### 2.4. Image Preprocessing and HAE Lesion Segmentation

T2WI data in DICOM format is uploaded to the Radcloud platform (version 3.1.0, http://radcloud.cn/, Huiying Medical Technology Co., Ltd., Beijing, China). As MR scanning with different field intensities is used, image preprocessing is required to obtain more robust radiomics features. Image preprocessing consists of two steps.

**Step 1 .**

We used the following formula to normalize the intensity of the image to minimize the change in MRI intensity collected by machines with different parameters (*i* is the original intensity; *F*(*i*) shows the normalized intensity; *μ*_*i*_ is the mean value of the image intensity values; *σ*_*i*_ indicates the standard deviation of the image intensity values; and *s* is an optional zoom and is set to 1 by default). Normalization is for the whole image, not just the region of segmentation.

(1) $$\begin{array}{c} F\left(i\right)=\frac{s\left(i-\mu_{i}\right)}{\sigma_{i}}. \end{array}$$

**Step 2 .**

In order to eliminate the intrinsic dependence of radiomics features on voxel size, the resampling method with a linear interpolation algorithm was used to normalize voxel size.

**Figure 2:**
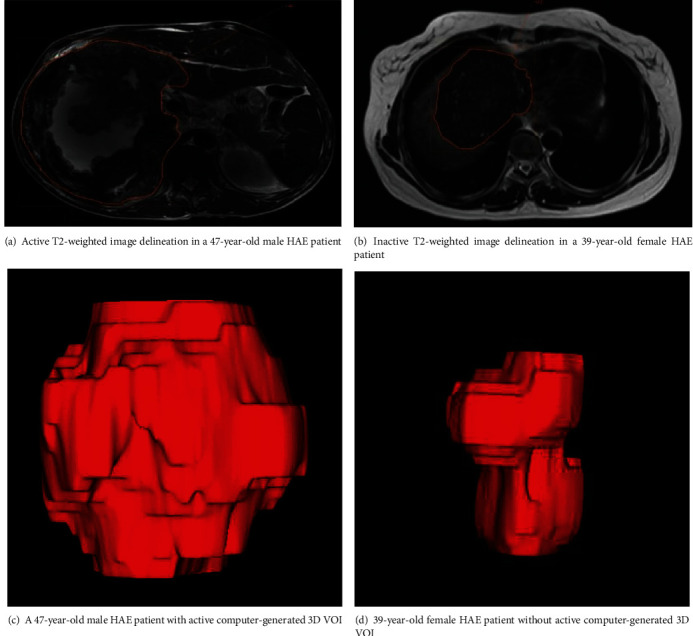
A representation of the manual segmentation in the T2-weighted images.

As shown in Figure 2, to obtain the volume of interest (VOI) for further analysis, four radiologists manually delineated the region of interest (ROI) along the edge of the lesion, layer by layer, on each T2WI. All depicted regions of interest (ROI) in T2WI were strictly delineated with the same criteria and visually validated by the same expert (with 10 years of experience in abdomen MRI). Then, the 3D VOI of the lesion is generated automatically by computer interpolation.

### 2.5. Feature Extraction

In this study, the “pyradiomics” package (version 2.1.2, https://pyradiomics.readthedocs.io/) in Python was used to extract 1409 radiomics features in the VOI of each T2WI. The features can be divided into four categories: shape features, first-order gray histogram features, second-order texture features, and higher-order features based on filter transformation. Shape features (*n* = 14) reflect the three-dimensional size and shape of a given VOI, including mesh volume, surface area, surface area to volume ratio, sphericity, compactness and spherical disproportion, elongation, and flatness. First-order gray histogram features (*n* = 18) reflect the overall information of the histogram, including energy, minimum, 10th/90th percentile, maximum, mean, median, standard deviation, range, mean absolute deviation, and entropy. Second-order texture features include the gray-level cooccurrence matrix (GLCM, *n* = 24), gray-level size zone matrix (GLSZM, *n* = 16), gray-level dependence matrix (GLDM, *n* = 14), neighborhood gray-level dependence matrix (NGLDM, *n* = 5), and gray-level run-length matrix (GLRLM, *n* = 16). The second-order texture features can respond to the image pixels at a certain level of relative distribution from the side, so they expound the complexity and heterogeneity within the lesion. High-order filter transform features (*n* = 1302) also include the original image through the filter transform to get the intensity and texture feature, through the neighborhood grayscale difference matrix and gray areas such as the size of the matrix computation, using seven kinds of filters: logarithm filter, exponential filter, gradient filter, square filter, square root filter, local binary pattern (LBP) filter, and wavelet filter. Features are compliant with definitions as defined by the Imaging Biomarker Standardization Initiative (IBSI) [19].

### 2.6. Subsampling

The experiment took into account the imbalance between the active and inactive HAE groups, which does not satisfy the balanced endpoint hypothesis of most machine learning-based prediction models. To tackle this problem, we use the synthetic minority oversampling technique (SMOTE) for subsampling. It is important to note, however, that the synthesized new data appears only in the training cohorts and not in the test cohorts.

### 2.7. Feature Selection and Model Construction

Prior to the steps of feature selection, because the range of radiomics features of different properties varies greatly, the normalization of radiomics features ensures the convergence of the training model. At the same time, in order to avoid model overfitting, fivefold cross-validation was performed during the experiments. In each fold of the training cohorts, the least absolute shrinkage and selection operator (LASSO) feature selection algorithm was used to select the relevant features and calculate the correlation coefficient of the selected features. The ten most valuable features with the highest correlation coefficient are retained as the final feature subset.

We used multiple ML algorithms to test the impact of different machine learning models on the predictive performance, including logistic regression (LR), multilayer perceptrons (MLP), and support vector machine (SVM). We developed diagnostic classifiers based on quantitative image grouping features (by using the T2WI features selected from the training queue) and quantitative radiomics features (by using the T2WI features selected in a training cohort). The evaluation indicators included the receiver operating characteristic curve (ROC curve) with indices of area under the curve (AUC), 95% confidence level (95% CI, AUC), accuracy, sensitivity, and specificity.

### 2.8. PET-CT Observation Items and Evaluation Criteria

The standardized uptake value (SUV) was calculated automatically by semiquantitative analysis in the workstation to judge the FDG uptake of the lesions according to the SUV value. Prior to analysis for measurement of SUV values, the basic information of each case and the location, size, and Kodama classification of the lesions were registered in detail to ensure that the lesions measured on PET-CT images and those segmented on MRI were one lesion. Most of the HAE lesions showed elevated cyclic glucose metabolism on PET-CT, and no hyperglycemia was found in the lesions. If the SUV of the lesion is higher than that of the surrounding liver at the same plane, the lesion is judged to be active; if the SUV value of the lesion is lower than that of the surrounding liver, the lesion is not active.

### 2.9. Statistical Analysis

The study was performed using the programming language Python 3.6 (https://www.python.org/). The packages of “pyradiomics” (https://pyradiomics.readthedocs.io/), “scikitlearn” (https://scikit-learn.org/), and “matplotlib” (https://matplotlib. org/) were used for feature selection, model building, and plotting in this study. Another statistical analysis was performed with SPSS 17.0 and MedCalc15.2.2. *P* value < 0.05 was considered statistically significant. ROC curve analysis was used to evaluate the diagnostic performances of ML classifiers.

## 3. Results

### 3.1. Demographic Data of the HAE Patients

Among the 136 patients, there were 64 males (47%) and 72 females (53%), with an average age of 39 ± 13 years. They were comprised of the Kazakh, Uygur, Han, and Tibetan ethnic groups. There was no significant difference in gender, age, lesion location, lesion size, and other clinical characteristics between the observation group and the control group (*P* > 0.05; Table 1).

### 3.2. Feature Selection of Radiomics

In this study, 1409 features were obtained from each T2WI VOI image, and the data were divided into five groups of different training and test sets using a fivefold cross-validation method (the data set was divided into 5 parts, 4 of which were training sets and 1 was a test set). The dimensionality of each training set was analyzed by the LASSO method, which adopted a 10-fold intragroup cross-validation strategy, and the maximum iteration times of the model. To avoid model overfitting problems caused by a high-feature dimension, an alpha with the least mean square error in cross-validation was selected, and the corresponding correlation coefficient was calculated. The absolute value of the correlation coefficient was further determined by ranking the correlation coefficients of features for groups with more than 10 features after LASSO feature screening. The 10 characteristics of HAE lesions are shown in Table 2.

**Figure 3:**
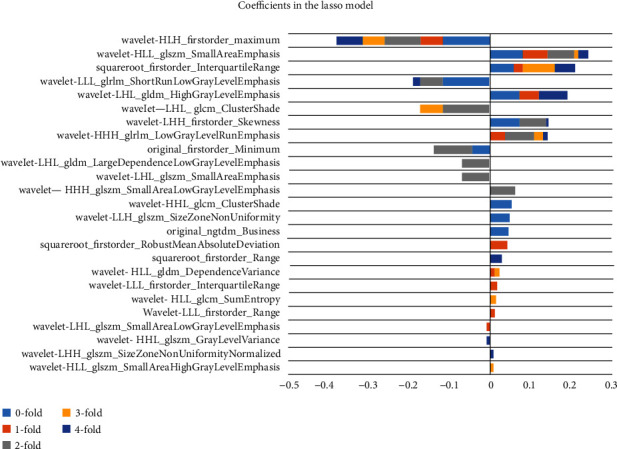
Cumulative graph of optimal feature coefficients of 5-fold cross-validation.

A bar graph was constructed using the sum of the best feature coefficients in the 5 groups of experiments with fivefold cross-validation, as shown in Figure 3. The results showed that optimal features were the first-order statistical features (*n* = 2) and texture features (*n* = 1) on the original image. Also, optimal first-order statistical features (*n* = 16) and texture features (*n* = 29) after wavelet transformation of the first-order statistical features of the maximum operator (wavelet-HLH_firstorder_Maximum) were obtained. The results suggested that cumulative maximum correlation coefficients can be used as biomarkers for effective radiomics to assess HAE characteristics.

### 3.3. Diagnostic Performance of the Radiomics Models

**Figure 4:**
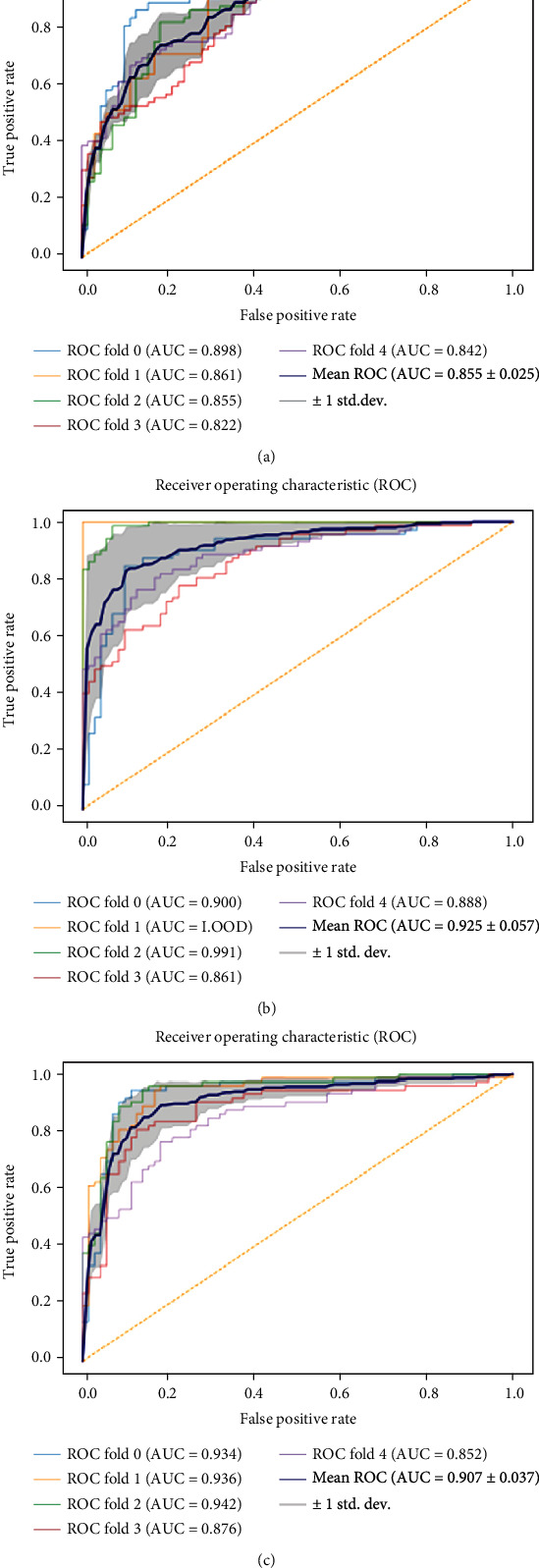
The ROC curves of the LR, MLP, and SVM machine learning classifiers in the training cohorts: (a) LR, (b) MLP, and (c) SVM.

**Figure 5:**
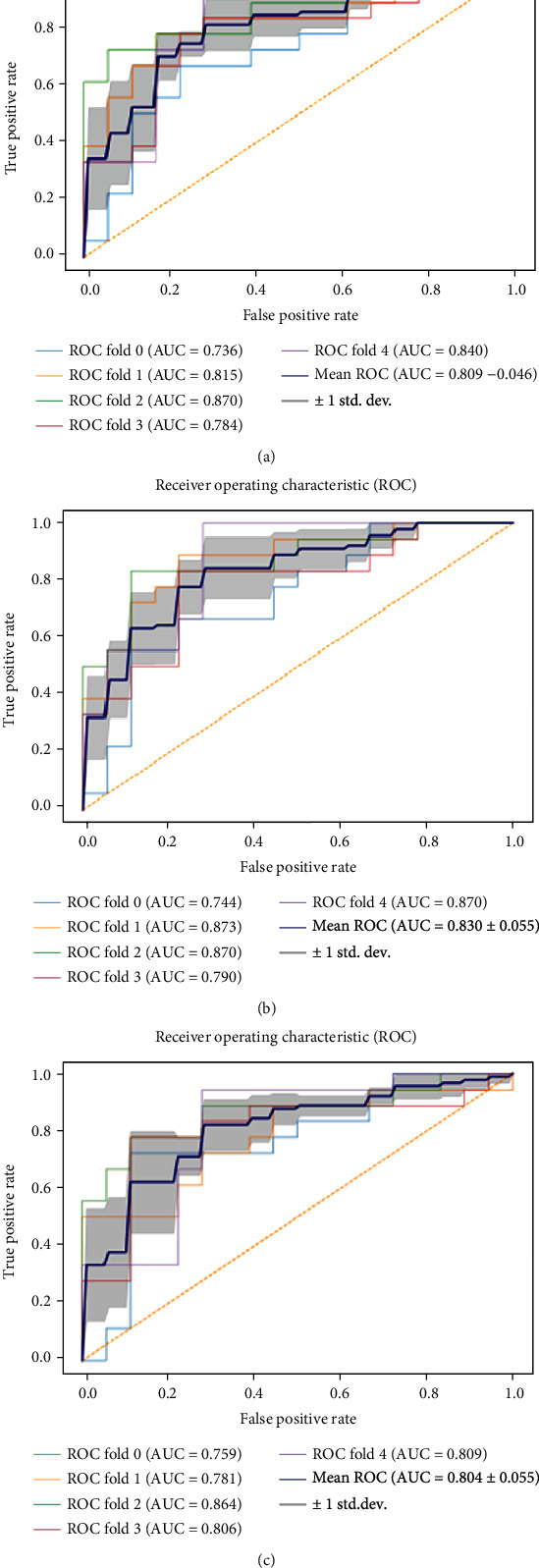
The ROC curves of the LR, MLP, and SVM machine learning classifiers in the test cohorts: (a) LR, (b) MLP, and (c) SVM.

A variety of machine learning algorithms were used to train the model using a fivefold cross-validation procedure. Figures 4 and 5 show the ROC curves of the three machine learning classifier models. Tables 3 and 4 summarize the diagnostic performance and model cutoff values of the three machine learning classifier models. In general, all three machine learning classifier models performed well. The average AUC of the test cohorts was higher than 0.800. The MLP had the best discrimination for HAE characteristic prediction. The mean AUC of the training cohorts was 0.925 ± 0.057, with a mean accuracy of 0.866. The mean sensitivity was 0.883, while the mean specificity was 0.889. Besides, the mean AUC of the test cohorts was 0.830 ± 0.053, with a mean accuracy of 0.817. The mean sensitivity was 0.822, and the mean specificity was 0.811.

## 4. Discussion

Hepatic alveolar echinococcosis (HAE) is a rare disease, often known as “worm cancer,” affecting the liver [20]. Compared with cystic echinococcosis (CE), HAE is by far more severe in affected patients. Despite the marked healthcare improvements in the western agricultural and pastoral regions of China, and the rising national health examination rates, the early detection rates of HAE cases continue to rise at an alarming rate [21, 22]. In the past, radical resection surgery was the first choice for patients with HAE [23]; however, with early diagnosis of lesions and the progress of treatment, choosing the treatment with less trauma and fewer complications can better reflect the humanistic care for the patients [24–26]. Therefore, it is imperative to accurately evaluate and analyze the biological activity of the lesions for better medical care. However, conventional imaging examinations are not sufficient to accurately and quantitatively evaluate the disease.

Invasive diagnostic methods, such as biopsy examinations, only obtain a small part of the lesion tissue, which may not fully reflect all characteristics of the lesion, and therefore, offer insufficient information. To date, a robust, noninvasive, affordable, and accessible method of evaluating and monitoring HAE lesions has not been developed. The rapid development of artificial intelligence, especially radiomics, in the field of radiology, in recent years, has presented unprecedented opportunities for the assessment of HAE lesions. Radiomics, which performs the high-throughput information extraction, yields robust and valuable data more reliably than visual observation. However, the research on its potential application in the diagnosis and treatment of HAE lesions is still in its initial stages.

The main pathological manifestations of HAE lesions are liquefaction, necrosis, calcification, and solid areas. Microscopically, numerous small vesicles are observed at the edge of the solid areas of the lesions. Many inflammatory cells, eosinophils, necrotic areas, new capillaries, and other structures are evident around the vesicles. The small vesicles continue to proliferate and erode the surrounding normal liver tissue. There are granulomatous reactions around HAE lesions characterized by fibrous tissue hyperplasia; infiltration of eosinophils, lymphocytes, and foreign body giant cells; and observable formation of alveolar echinococcosis nodules. This area is regarded as the bioactive part of HAE lesions. Therefore, internal vascularization and fibrosis of HAE lesions appear alternately and constitute the basic pathological changes of alveolar echinococcosis lesions in different periods [27–29].

Compared with CT and ultrasound images, MRI images can more accurately display the active part of the lesions, which can significantly reduce errors of manual operation for early imaging procedures. Besides, PET-CT has the characteristics of both anatomical morphology and functional metabolic imaging. It detects the status of glucose metabolism in parasites and indirectly interferes with the proliferative activity of lesions. The 18F-FDG PET-CT reflects the metabolism of the lesions through the semiquantitative index (SUV) value to determine biological activity in HAE lesions. Therefore, the 18F-FDG PET-CT shows the active areas of lesions that cannot be detected by traditional imaging examinations [30–32].

In this study, PET-CT showed that the FDG uptake pattern of HAE lesions was located in marginal areas, and most of them were semicircular and nodular, which was consistent with the distribution characteristics of small vesicles on MRI images. According to the study of Kodama et al. [33], in the early stage of HAE, a parasitic cyst manifests as a small vesicle structure. The formation of the germinal layer into a vesicle is among the two important larval development stages. Small vesicles structurally surround the granulation tissue, thus stimulating and mediating host immune responses [34]. The immune cells can absorb FDG, but the vesicles can not, thus indicating the biological activity of lesions. It could be better explained that 18F-FDG in PET-CT is mainly concentrated at the margin of the lesion rather than the small vesicles.

This study suggests that an imaging model based on the combination of radiomics features and machine learning methods might improve the accuracy of noninvasive diagnosis and serve as a valuable guide in clinical decision-making [35–37]. The construction of the HAE activity prediction model based on MRI radiomics features to evaluate the activity of HAE lesions does not use radiation, has high economic efficiency, and has high consistency with PET-CT, which would be an indispensable evaluation method for the diagnosis and treatment of HAE in the future.

In this study, conventional T2WI imaging features were extracted, and dimensionality reduction analysis was carried out by the LASSO regression algorithm to select the features that could best reflect the difference in HAE activity. The purpose of the LASSO method was to minimize the cost function and to obtain all features with nonzero coefficients, which would improve the interpretation and prediction accuracy of the model. The selected optimal feature subset contained a large number of first-order statistical features and texture features. The first-order statistical features reflect the internal voxel intensity of the lesions, and the texture features reflect the gray distribution characteristics in dimensional space, suggesting the heterogeneity of the lesions. Among them, the maximum intensity descriptor (wavelet-HLH_firstorder_Maximum, *P* = 0.00167, *U* test) appeared in five groups of experiments simultaneously showing the highest cumulative correlation. This indicates the heterogeneity of composition or distribution in HAE lesions by the maximum gray level intensity within the VOI and may be used as an effective imaging biomarker to evaluate the activity of HAE lesions.

Considering that the performance of some classifiers may vary with different lesions, we employed three machine learning methods with different computing mechanisms to construct a biological activity prediction model of HAE lesions. Both LR and SVM are linear classification algorithms if the kernel function is not considered. However, SVM only considers the points near the local boundary line, while LR considers all. MLP is a generalization of a single-layer perceptron, which could solve the nonlinear problems that a single-layer perceptron could not solve [38]. In this study, the MLP training cohorts showed a promising AUC of 0.928. Generally, the three models performed well, and the average AUCs of the test cohorts were higher than 0.800. This also suggests that MRI images have higher tissue resolution and could reflect the internal heterogeneity of the lesions better.

Also, the results showed significantly improved model sensitivity and specificity after the data ratio of the active group and the inactive group was balanced by the SMOTE algorithm. The SMOTE algorithm is an enhanced sampling method. Computation for new synthetic sampling is based on Euclidian distance for variables, rather than a simple oversampling [39]. It has been shown that SMOTE is robust to the variation of unbalanced ratio with various classifiers.

Nevertheless, the present research has several limitations. First, it is a single center study with a small sample size; further expanding the sample size and carrying out a multicenter study to improve the effectiveness of the model is needed. Second, the retrospective nature of this study, the long period, the incomplete clinical data, the manual segmentation of lesions, subjectivity, the inevitable existence of selective bias, and the results of different personnel calibration may affect the establishment of the model. Third, the radiomics model of HAE lesions based on MRI features needs further discriminant analysis with intrahepatic neoplasia and tumor with poor blood supply. Finally, the diagnostic efficiency of the radiomics model of HAE activity needs to be further compared with Kodama classification and Graeter classification.

## 5. Conclusions

In conclusion, T2WI-based imaging features and machine learning models can evaluate the biological activity of HAE lesions, which is helpful for the selection and monitoring of clinical treatment methods.

## Figures and Tables

**Table 1:** Demographic data of the HAE patients.

Patient attributes	Active group	Inactive group	*P* value
n	90	46	
Age (mean ± SD, yr)	39 ± 13	38 ± 14	0.847
Gender			0.816
Male	43	21	
Female	47	25	
Location of lesions			0.264
Less than 3 liver segments	18	14	
3-6 liver segments	67	28	
More than 6 liver segments	5	4	
Lesion size (mm^3^)	1388844.180	1357771.448	0.926

**Table 2:** 5-fold cross-validation for the best 5 group of best features.

Experimental group	0-fold	1-fold	2-fold	3-fold	4-fold
Feature name	wavelet-HLH_firstorder_Maximum	wavelet-HLL_glszm_SmallAreaEmphasis	wavelet-LHL_glcm_ClusterShade	squareroot_firstorder_InterquartileRange	wavelet-LHL_gldm_HighGrayLevelEmphasis
	wavelet-LLL_glrlm_ShortRunLowGrayLevelEmphasis	wavelet-HLH_firstorder_Maximum	original_firstorder_Minimum	wavelet-HLH_firstorder_Maximum	wavelet-HLH_firstorder_Maximum
	wavelet-HLL_glszm_SmallAreaEmphasis	wavelet-LHL_gldm_HighGrayLevelEmphasis	wavelet-HLH_firstorder_Maximum	wavelet-LHL_glcm_ClusterShade	squareroot_firstorder_InterquartileRange
	wavelet-LHH_firstorder_Skewness	squareroot_firstorder_RobustMeanAbsoluteDeviation	wavelet-LLL_glrlm_ShortRunLowGrayLevelEmphasis	wavelet-HHH_glrlm_LowGrayLevelRunEmphasis	squareroot_firstorder_Range
	wavelet-LHL_gldm_HighGrayLevelEmphasis	wavelet-HHH_glrlm_LowGrayLevelRunEmphasis	wavelet-HHH_glrlm_LowGrayLevelRunEmphasis	wavelet-HLL_gldm_DependenceVariance	wavelet-HLL_glszm_SmallAreaEmphasis
	squareroot_firstorder_InterquartileRange	squareroot_firstorder_InterquartileRange	wavelet-LHL_gldm_LargeDependenceLowGrayLevelEmphasis	wavelet-HLL_glcm_SumEntropy	wavelet-LLL_glrlm_ShortRunLowGrayLevelEmphasis
	wavelet-HHL_glcm_ClusterShade	wavelet-LLL_firstorder_InterquartileRange	wavelet-LHL_glszm_SmallAreaEmphasis	wavelet-HLL_glszm_SmallAreaEmphasis	wavelet-HHH_glrlm_LowGrayLevelRunEmphasis
	wavelet-LLH_glszm_SizeZoneNonUniformity	wavelet-LLL_firstorder_Range	wavelet-LHH_firstorder_Skewness	wavelet-HLL_glszm_SmallAreaHighGrayLevelEmphasis	wavelet-HHL_glszm_GrayLevelVariance
	original_ngtdm_Busyness	wavelet-HLL_gldm_DependenceVariance	wavelet-HLL_glszm_SmallAreaEmphasis		wavelet-LHH_glszm_SizeZoneNonUniformityNormalized
	original_firstorder_Minimum	wavelet-LHL_glszm_SmallAreaLowGrayLevelEmphasis	wavelet-HHH_glszm_SmallAreaLowGrayLevelEmphasis		wavelet-LHH_firstorder_Skewness

Note: glcm (gray-level cooccurrence matrix); glrlm (gray-level run-length matrix); glszm (gray-level-size zone matrix); gldm (gray-level dependence matrix); ngtdm (neighbouring gray-tone difference matrix).

**Table 3:** Diagnostic performance of machine learning-based MRI radiomics classifiers to assess the bioactivity of HAE lesions in the training cohort.

	AUC	Accuracy	Sensitivity	Specificity	95% CI, AUC	Cutoff
*LR*						
0-fold	0.898	0.868	0.861	0.875	0.836-0.942	0.517
1-fold	0.861	0.806	0.903	0.708	0.793-0.913	0.436
2-fold	0.855	0.819	0.819	0.819	0.786-0.908	0.484
3-fold	0.822	0.757	0.889	0.625	0.750-0.881	0.392
4-fold	0.842	0.778	0.708	0.847	0.772-0.898	0.563
Mean	0.855 ± 0.025	0.806	0.836	0.775	0.750-0.942	
*MLP*						
0-fold	0.900	0.875	0.847	0.903	0.839-0.944	0.544
1-fold	1.000	1.000	1.000	1.000	0.975-1.000	0.834
2-fold	0.991	0.958	0.986	0.931	0.958-1.000	0.387
3-fold	0.861	0.778	0.778	0.778	0.793-0.913	0.527
4-fold	0.888	0.819	0.806	0.833	0.825-0.935	0.488
*Mean*	0.925 ± 0.057	**0.886**	**0.883**	**0.889**	**0.793-1.000**	
*SVM*						
0-fold	0.898	0.868	0.861	0.875	0.836-0.942	0.517
1-fold	0.861	0.806	0.903	0.708	0.793-0.913	0.436
2-fold	0.855	0.819	0.819	0.819	0.786-0.908	0.484
3-fold	0.822	0.757	0.889	0.625	0.750-0.881	0.392
4-fold	0.842	0.778	0.708	0.847	0.772-0.898	0.563
Mean	0.907 ± 0.037	0.806	0.836	0.775	0.750-0.942	

**Table 4:** Diagnostic performance of machine learning-based MRI radiomics classifiers to assesses bioactivity of HAE lesions in the test cohort.

	AUC	Accuracy	Sensitivity	Specificity	95% CI, AUC
*LR*	0.809 ± 0.046	0.794	0.778	**0.811**	0.565-0.959
*MLP*	0.830 ± 0.053	**0.817**	**0.822**	**0.811**	**0.571-0.960**
*SVM*	0.804 ± 0.035	0.794	0.778	**0.811**	0.565-0.959

## Data Availability

The radiomics features data extracted from MR images are included within the supplementary information file. However, the image datasets in the current study are not publicly available due to patient privacy protection, but are available from the corresponding author on reasonable request.

## Abbreviations

ML: Machine learning
MRI: Magnetic resonance imaging
HAE: Hepatic alveolar echinococcosis
PET-CT: Positron emission tomography computed tomography
T2WI: T2-weighted images
SMOTE: Synthetic minority oversampling technique
LASSO: Least absolute shrinkage and selection operator
LR: Logistic regression
MLP: Multilayer perceptron
SVM: Support vector machine
ROC: Receiver operating characteristic curve
AUC: Area under the curve.
